# Swedish Translation and Cultural Adaptation of the Head and Neck Patient Symptom Checklist: An Instrument to Screen for Nutrition Impact Symptoms in Clinical Practice and Research

**DOI:** 10.1177/01939459241274342

**Published:** 2024-08-24

**Authors:** Ylva Tiblom Ehrsson, Sandra Einarsson, Per Fransson, Göran Laurell

**Affiliations:** 1 Department of Surgical Sciences, Section of Otorhinolaryngology and Head and Neck Surgery, Uppsala University, Uppsala, Sweden; 2 Department of Food, Nutrition and Culinary Science, Umeå University, Umeå, Sweden; 3 Department of Nursing, Umeå University, Umeå, Sweden

**Keywords:** translating, head and neck neoplasms, translation and cultural adaptation, nutrition impact symptoms, content validity, internal consistency

## Abstract

**Background:**

The Head and Neck Patient Symptom Checklist (HNSC) is a validated 2-part instrument used to ask patients with head and neck cancer about the nutrition impact symptoms they experience (part 1) and how these interfere with their eating (part 2).

**Purpose:**

The purpose of this work was to translate and culturally adapt the HNSC into Swedish in accordance with the guidelines of the International Society for Health Economics and Outcomes Research (ISPOR).

**Methods:**

The ISPOR guidelines include 10 steps, and these were thoroughly followed. In step 7, 9 health care professionals from the field of head and neck cancer assessed the perceived relevance (content validity) of each item in the HNSC, as well as the full HNSC. A total of 522 participants with head and neck cancer were included and followed up on 7 occasions using the HNSC to assess internal consistency.

**Results:**

The HNSC was translated from English into Swedish, ensuring accuracy through forward and backward translation and harmonization in the research team. Content validity for each part of the HNSC was rated excellent (scale content validity index 0.96). Internal consistency demonstrated a good Cronbach’s alpha score (>0.8) across the 7 follow-up time points (from baseline [before the start of treatment] and up to 24 months posttreatment).

**Conclusions:**

The HNSC has been successfully translated and culturally adapted into Swedish. The HNSC can be used in both clinical practice and research to screen for nutrition impact symptoms and symptoms that interfere with eating in patients with head and neck cancer.

**Trial registration:**

ClinicalTrials.gov NCT03343236 (date of registration: November 17, 2017)

Nutrition impact symptoms (NIS) are common in patients with head and neck cancer (HNC) and are sometimes present at diagnosis due to swallowing problems caused by the tumor, or later in the care trajectory due to the given single or multimodality treatment with radiotherapy, surgery, chemoradiotherapy, or other medical treatment that the patient receives.^1,2^ Common NIS resulting from treatment include chewing difficulties, swallowing problems, loss of taste, xerostomia, and oral pain.^3^ These NIS may cause problems with eating, impaired nutrition status, and aggravate existing comorbidities.^4,5^ NIS may also affect a patient’s quality of life (QOL) and psychological well-being, including in the long term.^6-8^ It is therefore particularly important to identify NIS before, during, and after treatment for HNC to properly support the patient in the health care system.

The Head and Neck Patient Symptom Checklist (HNSC) is a validated instrument, originally developed in Canada, and is used to ask patients about the NIS they experience and how these NIS interfere with their eating.^9,10^ The HNSC was developed based on a review of the literature^9^ and 12 of the NIS included in the instrument have also been used in a malnutrition screening and assessment tool called the *Scored Patient-Generated Subjective Global Assessment*.^11,12^ The HNSC has 2 parts and includes 17 NIS (pain, anxiety, dry mouth, loss of appetite, constipation, feeling full, depressed, thick saliva, diarrhea, sore mouth, lack of energy, nausea, difficulty chewing, smells bother me, vomiting, difficulty swallowing, and taste changes). When completing the instrument on paper, the patient circles a number on a 5-point Likert scale (1 = Not at all, 2 = A little bit, 3 = Somewhat, 4 = Quite a lot, 5 = A lot). In the first part of the HNSC, the patients rate how often they have experienced NIS (intensity) during the past 3 days and in the second part, they state if the NIS has interfered with their eating.^9,10^ In addition, as a free-text response, the patients can state if there are other symptoms they have experienced besides those that were predefined.

The questionnaire developers concluded that NIS play a crucial role in the nutrition surveillance of patients with HNC,^9^ especially those patients who are affected by diminished energy intake and weight loss. Using the HNSC to find and act on NIS may ultimately reduce the risk of malnutrition during treatment and follow-up among individuals diagnosed with HNC. Schmidt et al^10^ also argue that the instrument can be used to assess symptoms that disrupt the dietary habits of individuals with HNC. Hence, by utilizing the HNSC, health care professionals can proactively identify symptoms that might jeopardize the dietary intake, body weight, and functional capacity of patients with HNC.

No previous version of the HNSC has been translated and culturally adapted into the Swedish language. Thus, a translated version would be of great value for Swedish health care professionals. When an instrument is translated, it is important that the process is structured. For patient-reported outcome measures, the guidelines of the International Society for Health Economics and Outcomes Research (ISPOR) “Principles of Good Practice for the Translation and Cultural Adaptation Process for Patient-Reported Outcomes (PRO) Measures” have been proposed to guide the process.^13^

## Purpose

The purpose of this study was to describe the process of translating and culturally adapting the Head and Neck Patient Symptom Checklist (HNSC) from English into Swedish in accordance with the ISPOR guidelines.

## Methods

The ISPOR guidelines “Principles of Good Practice for the Translation and Cultural Adaptation Process for Patient-Reported Outcomes (PRO) Measures” include 10 steps^13^ and these were thoroughly followed.

### ISPOR Steps 1 to 6

In step 1, the preparation phase, permission to use and translate the HNSC was obtained by email from Dr C. Kubrak, who developed the HNSC, and also from Springer Science + Business Media, who published the original version of the instrument. The forward translation (step 2) into the native language (Swedish) was performed by the first author (YTE) and thereafter by another author (GL). This was followed by a reconciliation and consensus meeting with the whole research team (step 3) by comparing and merging the translations into a single version. In step 4, a back-translation into the original language (English) was performed by a bilingual translator. A review of the back translation (step 5) was conducted by comparing the back-translated version with the original version, followed by a harmonization meeting (step 6) with the whole research team in which a comparison of all translations and the original instrument was made to highlight whether there were any discrepancies between the various versions.

### ISPOR Step 7

Step 7 of the ISPOR process, the cognitive debriefing, was performed in 3 phases. In the first phase, we let a small group of nurses who were familiar with problems faced by patients with HNC review the translated Swedish version of the HNSC, which was followed by an informal discussion. In the second phase, experts in the field of HNC were asked to investigate the perceived relevance (content validity) of the HNSC instrument to screen for NIS in patients with HNC.^14^ In the third phase, internal consistency was measured to verify whether the Swedish version of the HNSC was fit for the intended purpose.^15^

#### Content validity

For phase 2 of the cognitive debriefing, experts (health care professionals) were invited to complete a questionnaire and rate the perceived relevance of each item in the Swedish version of the HNSC using a 4-point Likert scale (1 = very irrelevant, 2 = irrelevant, 3 = relevant, and 4 = very relevant). Two open-ended questions were used where the participants could freely address different aspects related to relevance and operational aspects of the HNSC. Demographic information was collected on current profession, area of clinical practice, and work experience (years). The data were collected over a period of 2 weeks from 2 hospitals.

#### Internal consistency

For phase 3 of the cognitive debriefing, data were used from a multicenter prospective observational study that started in October 2015 with data collection still ongoing (ClinicalTrials.gov no. NCT03343236). The prospective observational study was conducted according to the guidelines of the Declaration of Helsinki and was approved by the Regional Ethical Review Board in Uppsala (no. 2014/447). All patients gave their informed consent to participate. Inclusion criteria were newly diagnosed HNC patients with a curative treatment intent, ≥18 years old, and a performance status of 0 to 2 according to the Eastern Cooperative Oncology Group Performance Status/World Health Organization Performance Status.^16^ Exclusion criteria were previously treated malignant neoplasm within the past 5 years (except for skin cancer), inability to understand the Swedish language, or any cognitive impairment that could affect study compliance. The participants were enrolled before the start of treatment and followed up on a total of 7 occasions during the care trajectory up to 2 years after the end of treatment. The patients filled in the HNCS at the following 7 time points: baseline (after diagnosis and before the start of treatment), 4 and 7 weeks after the start of treatment, and 3, 6, 12, and 24 months after the end of treatment. Patients completed the Swedish version of the HNSC either in paper or digital format. In addition to data from the HNSC, information about the participants’ characteristics (ie, age, sex, tumor site, tumor stage, and treatment type) was used for the present study.

### Statistical Analyses

Responses about the perceived relevance from the experts in phase 2 were used to quantify content validity by calculating the content validity index (CVI). The CVI for each item of the HNSC was calculated (I-CVI) as well as the scale CVI (S-CVI) of the overall instrument.^10,17^ For the I-CVI, proportional scores ranging from 0 to 1 were generated by dividing the number of experts scoring 3 or 4 (agreed items) on the 4-point Likert scale by the total number of experts. The S-CVIs were calculated as the S-CVI average (S-CVI/Ave), that is, the S-CVI is an average of the I-CVIs. The I-CVI value for excellent content validity is predefined as 0.78 or higher.^14,17^ The S-CVI cutoff for acceptability was set at 0.80 and was considered excellent at 0.90.^17,18^

To quantify internal consistency in phase 3 of the cognitive debriefing step, Cronbach’s alpha coefficient was calculated at all 7 follow-ups for each item in the HNSC, as well as for the total score generated for all 17 items. Cronbach’s alpha was set at: excellent (α ≥ 0.9), good (0.8 ≤ α < 0.9), acceptable (0.7 ≤ α < 0.8), questionable (0.6 ≤ α < 0.7), poor (0.5 ≤ α < 0.6), and unacceptable (α < 0.5).^15^

Data were analyzed using the statistical software IBM SPSS version 28.0 (IBM). The participants’ characteristics were presented using descriptive statistics, that is, median (min, max), mean ± standard deviation (continuous variables), and numbers and percent (categorical variables).

## Results

### ISPOR Step 7

During the first phase of the cognitive debriefing, 2 research nurses reviewed the translated version of the instrument. This was followed by an informal discussion. From this first phase of cognitive debriefing, the nurses emphasized the need to clarify the text with information on how to fill in the instrument. The text was changed from: “Please circle the number that best describes how often you experienced the symptom during the past 3 days, and if it interfered with your eating” to: “Circle the number that best describes how often you experienced the symptom during the past 3 days (white column). If you have circled the numbers 2 to 5 in the white column, fill in the gray column regarding how the symptom has affected/interfered with your eating.”

#### Content validity

During the second phase, the 9 experts who completed the questionnaire were physicians (n = 5), registered nurses (n = 3), and 1 dietitian. They had been working in the field of HNC for a median of 19 years (min 5 years, max 30 years).

All items in the first part of the HNSC (intensity of NIS during the past 3 days) had excellent content validity, showing I-CVIs between 0.78 and 1.00 (Table 1). The full first part of the HNSC (intensity of NIS during the past 3 days) had excellent content validity (S-CVI 0.96).

**Table 1.** Item and Scale Indices for Content Validity (I-CVI, S-CVI) for the Head and Neck Patient Symptom Checklist, Part 1 (Intensity of Nutrition Impact Symptoms).

Item	Expert 1	Expert 2	Expert 3	Expert 4	Expert 5	Expert 6	Expert 7	Expert 8	Expert 9	No. of experts in agreement	I-CVI
Pain	√	√	√	√	√	√	√	√	√	9	1.00
Anxious	—	√	√	—	√	√	√	√	√	7	0.78
Dry mouth	√	√	√	√	√	√	√	√	√	9	1.00
Loss of appetite	√	√	√	√	√	√	√	√	√	9	1.00
Constipation	√	√	√	—	√	√	√	√	√	8	0.89
Feeling full	√	√	√	—	√	—	√	√	√	7	0.78
Depressed	√	√	√	√	√	√	√	√	√	9	1.00
Thick saliva	√	√	√	√	√	√	√	√	√	9	1.00
Diarrhea	√	√	—	√	√	√	√	√	√	8	0.89
Sore mouth	√	√	√	√	√	√	√	√	√	9	1.00
Lack of energy	√	√	√	√	√	√	√	√	√	9	1.00
Nausea	√	√	√	√	√	√	√	√	√	9	1.00
Difficulty chewing	√	√	√	√	√	√	√	√	√	9	1.00
Smells bother me	√	√	√	√	√	√	√	√	√	9	1.00
Vomiting	√	√	√	√	√	—	√	√	√	8	0.89
Difficulty swallowing	√	√	√	√	√	√	√	√	√	9	1.00
Taste changes	√	√	√	√	√	√	√	√	√	9	1.00
S-CVI/Ave:											0.96

The Head and Neck Patient Symptom Checklist (HNSC) has 17 items of nutrition impact symptoms (NIS) that patients with head and neck cancer may experience. The experts rated these items on a 4-point Likert scale (agreeing 3 or 4 = √). The first part of the HNSC is presented and shows the intensity of NIS, which assesses the symptom frequency during the last 3 days. I-CVI: Item content validity index (Number of experts agreeing [3 or 4 on the Likert scale] divided by the total number of experts. The I-CVI cutoff for excellent is ≥0.78). S-CVI/Ave: Scale content validity index (Average of the I-CVIs; the S-CVI cutoff for acceptability is ≥0.80 to 0.89 and is considered excellent at ≥0.90).

All items in the second part of the HNSC (if the NIS interfered with eating) had excellent content validity, showing I-CVIs between 0.78 and 1.00 (Table 2). The full second part of the HNSC (if the NIS interfered with eating) had excellent content validity (S-CVI 0.96).

**Table 2.** Item and Scale Indices for Content Validity (I-CVI, S-CVI) for the Head and Neck Patient Symptom Checklist, Part 2 (Nutrition Impact Symptom Interference).

Item	Expert 1	Expert 2	Expert 3	Expert 4	Expert 5	Expert 6	Expert 7	Expert 8	Expert 9	No. of experts in agreement	I-CVI
Pain	√	√	√	√	√	√	√	√	√	9	1.00
Anxious	√	√	√	—	√	√	√	√	√	8	0.89
Dry mouth	√	√	√	√	√	√	√	√	√	9	1.00
Loss of appetite	√	√	√	√	√	√	√	√	√	9	1.00
Constipation	√	√	√	√	√	—	√	√	√	8	0.89
Feeling full	√	√	√	√	√	—	√	√	√	8	0.89
Depressed	√	√	√	√	√	√	√	√	√	9	1.00
Thick saliva	√	√	√	√	√	√	√	√	√	9	1.00
Diarrhea	√	√	√	—	√	—	√	√	√	7	0.78
Sore mouth	√	√	√	√	√	√	√	√	√	9	1.00
Lack of energy	√	√	√	√	√	√	√	√	√	9	1.00
Nausea	√	√	√	√	√	√	√	√	√	9	1.00
Difficulty chewing	√	√	√	√	√	√	√	√	√	9	1.00
Smells bother me	√	√	√	√	√	√	√	√	√	9	1.00
Vomiting	√	√	√	√	√	—	√	√	√	8	0.89
Difficulty swallowing	√	√	√	√	√	√	√	√	√	9	1.00
Taste changes	√	√	√	√	√	√	√	√	√	9	1.00
S-CVI/Ave:											0.96

The Head and Neck Patient Symptom Checklist (HNSC) has 17 items of nutrition impact symptoms (NIS) that patients with head and neck cancer may experience. The experts rated these items on a 4-point Likert scale (agreeing 3 or 4 = √). The second part of the HNSC is presented and shows NIS interference, which assesses symptom interference with oral intake. I-CVI: Item content validity index (Number of experts agreeing [3 or 4 on the Likert scale] divided by the total number of experts. The I-CVI cutoff for excellent is ≥0.78). S-CVI/Ave: Scale content validity index (Average of the I-CVIs. The S-CVI cutoff for acceptability is ≥0.80 to 0.89 and is considered excellent at ≥0.90).

The health care professionals in phase 2 could also leave comments or suggestions regarding the HNSC. The following comments or suggestions were left by 3 professionals: (*a*) *Pain and sore mouth, they’re rather similar;* (*b*) *Many relevant symptoms and some of them are not something I would think of as a physician. It’s good to have a Likert scale from 1 to 5*; and (*c*) *Relevant questions.*

#### Internal consistency

The characteristics of the 522 participants included in phase 3 of the cognitive debriefing are presented in Table 3. Their mean age was 63.3 years (±11.3 years) with a male-to-female ratio of 2.3:1 (363 males). The most common tumor sites were the oropharynx (n = 216, 41.4%), oral cavity (n = 152, 29.1%), or larynx (n = 55, 10.5%). The treatment approaches were combined modality treatment (n = 293, 56.1%), single modality radiotherapy (n = 153, 29.3%), or single modality surgery (n = 76, 14.6%).

**Table 3.** Characteristics of 522 Included Participants With Head and Neck Cancer.

Characteristics	Subgroups	Value
Age, years, mean (±SD)		63.3 (±11.3)
Sex, n (%)	Male	363 (69.5)
	Female	159 (30.5)
Tumor site, n (%)	Oropharynx	216 (41.4)
	Oral cavity	152 (29.1)
	Larynx	55 (10.5)
	Cancer of unknown primary	27 (5.2)
	Nasopharynx	21 (4.0)
	Salivary glands	21 (4.0)
	Nasal and sinus	14 (2.7)
	Hypopharynx	10 (1.9)
	Other*	6 (1.1)
Tumor stage,^†^ n (%)	I	194 (37.2)
	II	115 (22.0)
	III	100 (19.2)
	IV	106 (20.3)
	Nonapplicable	7 (1.3)
Treatment type, n (%)	Combined modality treatment	293 (56.1)
	Single modality radiotherapy (RT)	153 (29.3)
	Single modality surgery	76 (14.6)

* Cancer of the external auditory canal (n = 3), ear cancer (n = 1), rhabdomyosarcoma in the left maxilla ethmoidal (n = 1), or lacrimal sac cancer (n = 1).

† According to version 8 of the Union for International Cancer Control (UICC).

The number of participants who filled in the HNSC at each follow-up (ie, baseline, 4 and 7 weeks after the start of treatment, and 3, 6, 12, and 24 months after the end of treatment) is presented in Table 4. On all 7 occasions, the HNSC contained some missing values as some patients chose to leave single items blank.

**Table 4.** Number of Participants Filling in the Head and Neck Patient Symptom Checklist at Each Follow-Up.

Follow-up	Total no. who completed the HNSC	Filled in the HNSC digitally	Filled in the HNSC by paper	Missing single item/s	Total no. filling in all single items	Deceased	Missing*
Baseline, before the start of treatment	505	216	289	27	478	—	17
4 weeks after the start of treatment	416	156	260	16	400	4	102
7 weeks after the start of treatment	454	196	258	27	427	—	64
3 months after the end of treatment	450	201	249	26	424	7	61
6 months after the end of treatment	415	215	200	27	388	13	83
12 months after the end of treatment	381	195	186	25	356	16	101
24 months after the end of treatment	285	138	147	9	276	34	163

Abbreviation: HNSC: Head and Neck Patient Symptom Checklist.

* Due to dropping out or had not reached the follow-up by this time.

The Cronbach’s alpha for each NIS item and the total score for all 17 items at the 7 follow-ups are presented in Table 5. The total score for all 17 items was “Good” (Cronbach’s alpha between 0.8 ≤ α < 0.9) with the total Cronbach’s alpha score values at baseline 0.84, 4 weeks after the start of treatment 0.84, 7 weeks after the start of treatment 0.86, 3 months after the end of treatment 0.81, 6 months after the end of treatment 0.83, 12 months after the end of treatment 0.82 and 24 months after the end of treatment 0.84. It was not possible to state the Cronbach’s alpha for NIS interfering with eating (second part of the HNSC) as there were too few values.

**Table 5.** Cronbach’s Alpha on Nutrition Impact Symptoms From the Head and Neck Patient Symptom Checklist.

NIS	Baseline, before the start of treatment, n = 478		4 weeks after the start of treatment, n = 400		7 weeks after the start of treatment, n = 427		3 months after the end of treatment, n = 424		6 months after the end of treatment, n = 388		12 months after the end of treatment, n = 356		24 months after the end of treatment, n = 276	
	Scale variance	α	Scale variance	α	Scale variance	α	Scale variance	α	Scale variance	α	Scale variance	α	Scale variance	α
Pain	65.12	0.82	101.54	0.83	121.48	0.84	73.15	0.79	70.69	0.81	64.84	0.80	67.82	0.83
Anxious	70.54	0.84	106.73	0.83	130.48	0.85	76.05	0.80	72.18	0.81	66.35	0.81	68.97	0.83
Dry mouth	69.54	0.83	101.15	0.83	126.16	0.85	74.56	0.80	69.71	0.82	65.88	0.82	65.84	0.83
Loss of appetite	67.83	0.82	96.75	0.82	120.70	0.85	71.83	0.79	70.34	0.81	66.73	0.81	66.83	0.82
Constipation	72.38	0.83	105.09	0.83	128.77	0.86	78.49	0.80	76.31	0.82	69.66	0.82	69.89	0.83
Feeling full	70.92	0.84	107.59	0.84	134.42	0.86	77.13	0.81	73.23	0.82	68.10	0.82	67.61	0.83
Depressed	72.91	0.84	109.81	0.84	134.43	0.86	77.13	0.80	73.39	0.81	67.93	0.81	70.29	0.83
Thick saliva	70.14	0.83	100.02	0.83	122.92	0.85	76.27	0.81	71.43	0.82	66.03	0.81	65.59	0.83
Diarrhea	76.15	0.84	111.32	0.84	135.64	0.86	81.87	0.81	78.19	0.83	71.90	0.82	73.34	0.84
Sore mouth	64.83	0.82	97.91	0.82	118.02	0.84	74.30	0.80	70.05	0.81	65.39	0.80	67.58	0.83
Lack of energy	69.13	0.83	102.98	0.83	123.41	0.85	72.89	0.79	69.23	0.81	65.00	0.81	66.84	0.83
Nausea	74.41	0.83	104.95	0.83	127.75	0.85	81.12	0.80	77.59	0.82	71.34	0.81	73.10	0.83
Difficulty chewing	67.00	0.82	105.97	0.84	123.88	0.85	74.93	0.80	69.14	0.81	64.99	0.81	65.85	0.83
Smells bother me	74.43	0.84	106.66	0.83	129.99	0.86	78.00	0.80	75.25	0.82	69.93	0.82	70.97	0.84
Vomiting	77.24	0.84	110.67	0.84	131.82	0.86	83.80	0.81	80.72	0.83	73.54	0.82	75.44	0.84
Difficulty swallowing	67.46	0.83	101.61	0.83	122.36	0.85	74.67	0.80	69.76	0.81	64.96	0.81	65.38	0.82
Taste changes	71.13	0.83	98.78	0.83	121.88	0.85	72.17	0.80	70.62	0.82	65.61	0.81	67.53	0.84
Total for all 17 items	N/A	0.84	N/A	0.84	N/A	0.86	N/A	0.81	N/A	0.83	N/A	0.82	N/A	0.84

Nutrition Impact Symptom (NIS) intensity was assessed on a 5-point Likert scale (1 = not at all; 5 = a lot) during the last 3 days. From the start of treatment until follow-up 24 months after the end of treatment.

### ISPOR Steps 8 to 10

A review of the cognitive debriefing results was performed and discussed by the research team (step 8). This step resulted in one change being made to the instrument. For the term “depressed,” 2 Swedish words were used to describe the same term. In step 9, the final version of the instrument was checked for spelling and/or grammatical errors by a proofreader who had not previously been involved in the translation process. Step 9 resulted in 9 minor changes being made to the instrument (eg, uppercase consistency, added comma characters, and hyphens). Step 10, a report on each step of the translation and cultural adaptation of the instrument, is completed through the publication of this study in a peer-reviewed scientific journal.

## Discussion

The aim of this work was to develop a Swedish version of the HNSC by following the 10 steps of the ISPOR guidelines.^13^ The guidelines are an established method that help ensure that the translation and cultural adaptation of patient-reported outcome measures adhere to a set standard, allowing for the meaningful and valid measurement of patient-reported outcome measures across different languages and cultures. The HNSC was well-translated from the original language (English) into Swedish and no significant linguistic issues were detected. However, creating a culturally adapted version of an instrument generally involves more than just mere translation.^19^ For example, during the cognitive debriefing, we realized that we needed to provide a more detailed and thorough explanation of how to fill in the instrument as this was not clear from the more straightforward translation. Another important step is harmonization, which was achieved by the multidisciplinary nature of the research team, comprising 2 registered nurses, 1 physician, and 1 registered dietitian, all with extensive experience of HNC, from both clinical practice and research.

For the health care professionals, the I-CVI values and the S-CVI/Ave were all above the suggested cutoff scores, for both the items on NIS intensity and the items on NIS interfering with eating. For the items on NIS intensity, 2 symptoms, *anxiety* and *constipation*, had I-CVI values of 0.78, which is the minimum acceptable threshold for achieving excellent content validity. In addition, for the items on NIS interfering with eating, one symptom, *diarrhea*, achieved the same value of 0.78. These 3 symptoms are not directly associated with problems in the upper digestive tract when compared to the other NIS in the HNSC, which could explain the lower score of I-CVI by the health care professionals. Nevertheless, these 3 symptoms are included in other instruments that screen for NIS in patients with HNC^20,21^ and have been found to be of importance for selected groups of patients with HNC.^20,22-24^ For example, previous studies have shown that anxiety can be a problem for patients at the beginning of their treatment trajectory^20^ and that patients treated with a multimodal approach, for example, chemoradiotherapy, might be more exposed to problems related to the gastrointestinal tract.^23^ Due to the complex pain experienced by patients with HNC, opioids are often used, which can lead to constipation^24^ which has been shown to be a symptom more frequently present in malnourished patients with HNC.^22^ In addition, Kubrak et al^9^ demonstrated that the HNSC was capable of identifying NIS in 52 patients treated for HNC.

Internal consistency measured using Cronbach’s alpha is the most extensively utilized measure for assessing the reliability of a scale in various fields of research and measurement.^25^ This index provides a comprehensive evaluation of internal consistency, offering valuable insights into the degree to which the items on a scale consistently measure the same underlying construct. The results of the present study of 522 patients with HNC show good values of the Cronbach’s alpha for the total of 17 NIS items from 0.86 to 0.81 across the 7 follow-ups. This can be compared to Schmidt et al,^10^ who used the HNSC in adult patients treated for HNC. Their validation included 368 patients who filled in the HNSC and they found sensitivity to be 79% to 98%, specificity 99% to 100%, positive predictive value 92% to 100%, and negative predictive value 94% to 100%. The Cronbach’s alpha was 0.92.^10^ The HNSC has also been translated from English into Chinese in a study of 116 patients with HNC undergoing radiotherapy.^26^ The results for the Cronbach’s alpha were 0.79 for the total of 17 NIS items and 0.80 for NIS items interfering with eating, respectively.^26^

### Implications for Clinical Practice

The translated and culturally adapted HNSC is now available for Swedish clinical HNC settings. Kubrak et al^9^ and Schmidt et al^10^ describe the HNSC as an instrument that can be used in clinical practice at initial assessment to gather comprehensive information about patient symptoms, their severity, and their impact on daily functioning. The HNSC is also described as an instrument that can be used in different health care settings, such as identifying and documenting specific symptoms during and after treatment and evaluating rehabilitation measures. The HNSC can also facilitate communication between health care providers and patients on symptom management strategies as it allows patients to describe their symptoms in a structured way, ensuring that their concerns are addressed, thereby promoting shared decision-making and patient-centered care. Hence, the information gained can assist health care professionals in developing an appropriate treatment plan tailored to the individual needs of the patient. This is particularly important for this multifaceted group of patients, who are often left with unmet needs when in contact with the health care system.^27,28^ By understanding the specific symptoms and their severity, clinicians can prioritize interventions and set realistic goals for symptom management (eg, relative to body weight loss and dietary intake). The HNSC can also be used to track changes in symptoms over time.^9,10^ By periodically repeating the HNSC, clinicians can assess the effectiveness of interventions to reduce symptoms that are disabling for the patient and also measure rehabilitative outcomes. This longitudinal assessment helps monitor the patient’s progress, identify any new or worsening symptoms, and ensure timely interventions.

### Strengths and Limitations

The patients who completed the HNSC were recruited from 3 different tertiary referral centers in Sweden, allowing a multicenter approach to be taken to the data collection. The patients were also followed up on 7 occasions using the HNSC—before, during, and up to 2 years after the end of treatment—resulting in repeated use of the instrument. A total of 522 patients were enrolled in the study, and although not all of them consistently completed the HNSC at each follow-up, a noteworthy sample size was achieved. Despite the absence of established criteria for this kind of study, a sample size of at least 50 to 100 participants is generally recommended.^29^ A potential limitation of our work is the presence of selection bias among both health care professionals and patients, as they were not recruited consecutively. This nonconsecutive recruitment approach may introduce a bias in the sample, as those patients who participated may not be fully representative of the broader population under investigation. Furthermore, according to our study’s inclusion criteria, all the enrolled patients exhibited a performance status ranging from 0 to 2 based on the Eastern Cooperative Oncology Group Performance Status/World Health Organization Performance Status.^16^ This meant that we excluded those individuals with limited self-care ability who spent over 50% of their waking hours confined to a bed or chair, as well as those individuals who were completely disabled, unable to perform any self-care, and entirely bed or chair-bound.

## Conclusion

Aligning with the ISPOR process, the HNSC has been successfully translated and culturally adapted into a Swedish version that can now be used in both clinical practice and research to screen for NIS intensity and NIS that interfere with eating in patients with HNC. The result shows that the Swedish version of the HNSC received an excellent rating on content validity, confirming the instrument’s relevance in screening for NIS in patients with HNC. Internal consistency confirmed the reliability of the instrument’s 17 NIS items. The HNSC has major clinical implications and can be used in different health care settings to identify and document specific NIS during and after treatment and evaluate rehabilitation measures, facilitating patient-centered care, and communication.
